# Innate Immune Responses in Pediatric Patients with Gastritis—A Trademark of Infection or Chronic Inflammation?

**DOI:** 10.3390/children9020121

**Published:** 2022-01-18

**Authors:** Lorena Elena Meliț, Cristina Oana Mărginean, Maria Oana Săsăran, Simona Mocanu, Dana Valentina Ghiga, Adriana Crișan, Claudia Bănescu

**Affiliations:** 1 Department of Pediatrics I, George Emil Palade University of Medicine, Pharmacy, Science, and Technology of Târgu Mureș, Gheorghe Marinescu Street No. 38, 540136 Târgu Mureș, Romania; lory_chimista89@yahoo.com; 2 Department of Pediatrics III, George Emil Palade University of Medicine, Pharmacy, Science, and Technology of Târgu Mureș, Gheorghe Marinescu Street No. 38, 540136 Târgu Mureș, Romania; oanam93@yahoo.com; 3 Department of Pathology, County Emergency Clinical Hospital of Târgu Mureș, Gheorghe Marinescu Street No. 50, 540136 Târgu Mureș, Romania; slmocan70@gmail.com; 4 Department of Scientific Medical Research Methodology, George Emil Palade University of Medicine, Pharmacy, Science, and Technology of Târgu Mureș, Gheorghe Marinescu Street No. 38, 540136 Târgu Mureș, Romania; valentinaghiga@gmail.com; 5 Department of Genetics, Center for Advanced Medical and Pharmaceutical Research, George Emil Palade University of Medicine, Pharmacy, Science, and Technology of Târgu Mureș, Gheorghe Marinescu Street No. 38, 540136 Târgu Mureș, Romania; adriana.cosma17@gmail.com (A.C.); claudia.banescu@gmail.com (C.B.)

**Keywords:** innate immune response, children, gastritis, TLR2 rs3804099 gene polymorphisms, TLR2 rs3804100 gene polymorphisms, NLRP3 rs10754558 gene polymorphisms

## Abstract

The aim of this study was to define the relationship between several environmental, laboratory, and genetic factors, i.e., TLR2 and NLRP3 polymorphisms, and *Helicobacter pylori* (*H. pylori*) infection in children, by comparing three different groups of pediatric subjects: *H. pylori*-induced gastritis, non-*H. pylori* gastritis, and healthy controls. Our final study sample included 269 children, which were divided into three groups according to the histopathological exam: group 1 with 51 children with *H. pylori*-induced gastritis, group 2 with 103 children with *H. pylori*-negative gastritis, and group 3 (control group) with 115 children without any histopathological changes. All children underwent a thorough anamnesis, clinical exam, laboratory tests, and upper digestive endoscopy with gastric biopsy for rapid urease test, histopathological exam, and genetic analysis of *TLR2* rs3804099, *TLR2* rs3804100, and *NLRP3* rs10754558 gene polymorphisms. We noticed a significant association between living conditions and the type of gastritis (*p* < 0.0001). Both rapid urease and serological tests were significantly associated with the presence of *H. pylori* (*p* < 0.0001). The CT variant genotype of *TLR2* rs380499 was significantly associated with neutrophil count (*p* = 0.0325). We noticed a significant association between the CC variant genotype of *NLRP3* rs10754558 and leucocytes, neutrophils, eosinophils, as well as ALT (*p* = 0.0185, *p* = 0.0379, *p* = 0.0483, *p* = 0.0356). Based on these findings, we state that poor living conditions and rural areas represent risk factors for *H. pylori* infection. The rapid urease test is a reliable diagnostic tool for this infection. CT and TT carriers of *TLR2* rs3804099, as well as CC carriers of *NLRP3* rs10754558, might display a more severe degree of systemic inflammation.

## 1. Introduction

Chronic inflammation is the hallmark of carcinogenesis, playing a crucial role in the development of a wide diversity of solid tumors. According to the World Health Organization, *Helicobacter pylori* (*H. pylori*) is a class 1 carcinogen, infecting more than 50% of the world’s population. It is well-documented that this infection might lead to chronic gastritis, peptic ulcers, gastric cancer, and mucosa-associated lymphoid tissue lymphomas [1,2]. It was previously demonstrated that *H. pylori*-associated inflammation is closely related to the host’s innate immune system since it is capable of triggering these responses via particular pathogenicity features such as cag-pathogenicity island (cagPAI) and vacuolating cytotoxin A (VacA), or pathogen-associated molecular patterns (PAMPs), involving flagellin and lipopolysaccharides (LPS) [3,4]. The combination of PAMPs, toll-like receptors (TLRs), and nucleotide-binding oligomerization domain (NOD)-like receptors (NLRs) results in the activation of immune cells receptors and subsequent promotion of both secretion and expression of several proinflammatory cytokines. Furthermore, *H. pylori* has the ability to upregulate IL-1β and IL-18 in human immune cells and gastric tissue of animal models, proving its impact on the activation of inflammasomes [5,6,7].

Inflammasomes belong to the innate immune system and represent multimeric protein complexes triggered by several inflammatory stimuli. Thus, the NOD-like receptor family pyrin domain-containing 3 (NLRP3) is an important complex reported to participate in the pathogenesis of several chronic inflammatory diseases such as arthritis and colitis, but its increased expression was also reported in gastric cancer [8,9,10]. It was shown that in the setting of *H. pylori* infection, macrophages and dendritic cells produce IL-1β, which further activates NLRP3. The latter promotes local neutrophil infiltration, an important inflammatory reaction that decreases the secretion of gastric acid with a significant impact on the long-term survival of bacteria in the gastric mucosa [11,12]. Contrariwise, studies reported that the inhibition of caspase-1, a crucial player in the maturation of IL-1β and IL-18, reduces gastric inflammation by blocking the NLRP3 inflammasome pathway [13]. The activation of NLRP3 is a two-step process, consisting initially in the transcription of pro-IL-1β and NLRP3 as a result of NF-kB and AP-1 activation by pattern-recognition receptors (PRRs) in response to microbial stimuli, and secondly in the oligomerization of NLRP3 inflammasome, activation of caspase-1 and pro-IL-1β cleavage by caspase-1, and finally the secretion of mature IL-1β [14,15].

It is well-established knowledge that TLRs, another crucial component of the innate immune system, are involved in the regulation of both innate and adaptive immune responses through the recognition of diverse microbial products such as peptidoglycans, lipoproteins, and LPS [3]. Moreover, it was emphasized that surface TLRs such as TLR1, 2, 4, 5, and 6 are able to recognize surface bacterial components, while TLR3, 7, 8, and 9 participate in recognition of microbial nucleic acids [3]. Furthermore, the signaling cascades induced by these TLRs are essential for adaptive immune responses by inducing multiple costimulatory molecules [16]. TLRs genetic variation by specific single-nucleotide polymorphisms might influence the host’s immune response by increasing or decreasing the susceptibility to *H. pylori* infection. Thus, these receptors are responsible for enabling *H. pylori* prolonged persistence within the gastric mucosa and for promoting chronic inflammation with subsequent carcinogenesis [17,18,19]. TLR2 acts in collaboration with TLR4 to recognize *H. pylori* LPS, both being activated in cooperation with the adapter molecule MyD88, which further promotes the mitogen-activating protein kinase (MAPK) signaling pathway. All these processes eventually result in the activation of the transcription of nuclear factor (NF)-κB, which causes a severe inflammatory response characterized as chronically active gastritis with increased oncogenic potential by stimulating the immediate expression of inducible nitric oxide synthetase (iNOS), proinflammatory cytokines, chemokines, and their receptors, as well as interleukins [20].

Considering that *H. pylori* is commonly acquired during childhood, the detection of factors that enable its prolonged persistence within the gastric mucosa in pediatric patients might represent the cornerstone in the prevention of *H. pylori*-related carcinogenesis. Thus, the aim of this study was to define the relationship between several environmental, laboratory, and genetic factors, i.e., TLR2 and NLRP3 polymorphisms, and *H. pylori* infection in children, by comparing three different groups of pediatric subjects: *H. pylori*-induced gastritis, non-*H. pylori* gastritis, and healthy controls.

## 2. Materials and Methods

### 2.1. Study Sample

Our cross-sectional prospective study included 280 children admitted to the Pediatrics Clinic 1 Târgu Mureş, Romania, between March 2016 and July 2021, who presented for dyspeptic symptoms (e.g., abdominal or epigastric pain, nausea, vomiting, heartburn, etc.), with ages between 1 and 18 years, and no history of chronic disorders or recent infectious disease.

All children who complained of dyspeptic symptoms underwent upper digestive endoscopy, under the condition that their parents or caregivers agreed and signed the informed consent. The exclusion criteria consisted of children under one year of age due to the characteristics of the video endoscope, abnormal clinical exam or laboratory parameters (e.g., fever, leukocytosis, positive acute phase reactants, etc.), the parents’/caregivers’ refusal to sign the informed consent for the inclusion of their children in the study, and those with incomplete data. Based on the aforementioned criteria, we excluded 11 children from the study: 10 with abnormal laboratory parameters and one due to parents’ refusal to sign the informed consent. All the pediatric patients included in this study benefited from a clinical exam and thorough anamnesis based on the statements of their parents/caregivers. Good living conditions were defined as no history of bed-sharing or living in crowded places, a proper economic level, and adequate hygiene habits. Thus, all parents/caregivers were asked basic questions regarding their living conditions (e.g., originating area, number of family members, type of water source, estimated income, aspects regarding hygiene habits, or bed-sharing between adults and children). These questions were chosen based on the previously reported risk factors for *H. pylori* infection. We took a blood sample from each child in order to assess several laboratory parameters such as complete cellular blood count, C-reactive protein (CRP), erythrocyte sedimentation rate (ESR), liver enzymes—alanine aminotransferase (ALT), aspartate aminotransferase (AST), gamma-glutamyl transpeptidase (GGT)—serum iron, and neutrophils/lymphocytes ratio (NLR), which was computed by dividing the neutrophils to the lymphocytes. Further, we assessed the gastric mucosa of all children endoscopically, and we obtained five biopsy samples: two (one from the antrum and one from the gastric) for the rapid urease tests, two biopsies from the gastric antrum for the histopathological exam, and one biopsy from the gastric antrum for the genetic analysis, which was collected in an Eppendorf tube filled with a stabilization solution in order to avoid the immediate need for DNA isolation and purification in the genetics laboratory. The upper digestive endoscopies were performed using a single trained physician with experience in pediatric gastroenterology. The histopathological exam used Giemsa staining for the detection of *H. pylori*. Moreover, the characterization of gastritis type was performed according to the Sydney classification, considering the type of the inflammatory infiltrate [21], and was thoroughly assessed by a single experienced pathologist. The presence of gastroesophageal and biliary reflux was established, taking into consideration anamnesis, clinical exam, and endoscopic findings.

### 2.2. Ethics

The study was performed according to the principles of the Helsinki declaration, and it was approved by the Ethics Committee of the University of Medicine, Pharmacy, Sciences, and Technology ‘George Emil Palade’ Târgu Mureş (No. 27/17 March 2016, and No. 792/11 March 2020). Moreover, we obtained the signed informed consent of all the parents/caregivers and the assent of all children prior to their inclusion in the study.

### 2.3. Genotyping Analysis

The extraction of genomic DNA (gDNA) from fresh gastric tissue samples obtained by upper digestive endoscopies was performed using the PureLink Genomic DNA Mini Kit (from ThermoFischer Scientific, Waltham, MA, USA). We obtained gDNA according to the manufacturer’s protocol, which was further quantified using an Eppendorf BioSpectrometer (from Eppendorf, Wien, Austria GmbH). The TLR2 rs3804099, TLR2 rs3804100 and NLRP3 rs10754558 gene polymorphisms were assessed using TaqMan technology and predesigned assay namely, C__22274563_10, C__25607727_10, C__26052028_10 on an Applied Biosystems™ 7500Fast Dx Real-Time PCR System. For both TLR2 rs3804099 and TLR2 rs3804100 gene polymorphisms, the C allele was considered a variant with CC and CT as variant genotypes, while for NLRP3 rs10754558, we also defined the C allele to be variant with CC and CG as variant genotypes.

### 2.4. Statistical Analysis

The statistical analysis consisted of both descriptive (frequency, percentage standard deviation, mean, median) and inferential statistics elements. The Shapiro–Wilk test was used for determining the distribution of the analyzed series. The ANOVA, Kruskal–Wallis, and Dunn’s Multiple Comparison tests were applied for the comparison of means and medians. We also used the Chi-squared test for identifying the association between the qualitative variables. The significance threshold for the *p* value was chosen at 0.05. The statistical analysis was performed using the GraphPad Prism trial version.

## 3. Results

### 3.1. The Demographic Analysis of the Sample

**Figure 1:**
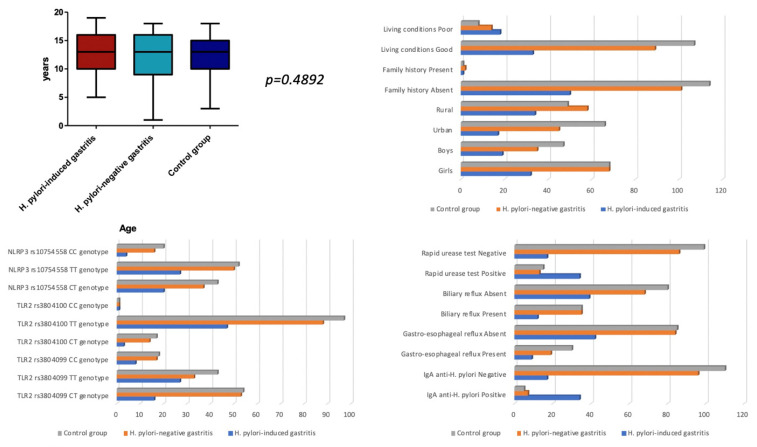
The demographic analysis of the groups included in the study.

Our final sample consisted of 269 children divided into three groups according to the histopathological exam. In group 1 were 51 children with *H. pylori*-induced gastritis; in group 2, 103 children with *H. pylori*-negative gastritis, and in group 3, the control group, were 115 children without any histopathological changes. We found a similar mean age between the three groups (*p* = 0.4892), with a slight predominance of female gender in group 3 (*p* = 0.5764). Regarding the originating area, we observed that children with gastritis, irrespective of the presence of *H. pylori*, were mostly living in rural areas (*p* = 0.0100). Regarding the living conditions, we noticed a significant association between living conditions and the type of gastritis (*p* < 0.0001), indicating that poor living conditions predominated in the group with *H. pylori*-induced gastritis (35.29%), followed by the group with other types of gastritis (13.59%) versus only 6.96% in the control group. Our findings also revealed that both rapid urease and serological tests were significantly associated with the presence of *H. pylori* in the histopathological exam (*p* < 0.0001). We found no significant differences between the three groups in terms of TLR2 rs3804099, TLR2 rs3804100, and NLRP3 rs10754558 gene polymorphisms (*p* = 0.1339, *p* = 0.5971, and *p* = 0.5570), family history (*p* = 0.7700), gastroesophageal reflux (*p* = 0.2944), and biliary reflux (*p* = 0.4151). All assessed parameters are described in Table 1 and Figure 1.

### 3.2. The Laboratory Parameters of the Three Groups

Regarding the assessed laboratory parameters, no significant differences were observed between the three groups (Table 2), except for CRP, which varied significantly among the three groups (*p* = 0.0452), being significantly higher in *H. pylori*-negative gastritis group versus the control group, *p* = 0.0232 (Table 3 and Table 4). We must mention that we excluded from each group the children who benefited from a qualitative assessment of CRP.

### 3.3. TLR2 rs380499 Gene Polymorphism and Laboratory Parameters

**Figure 2:**
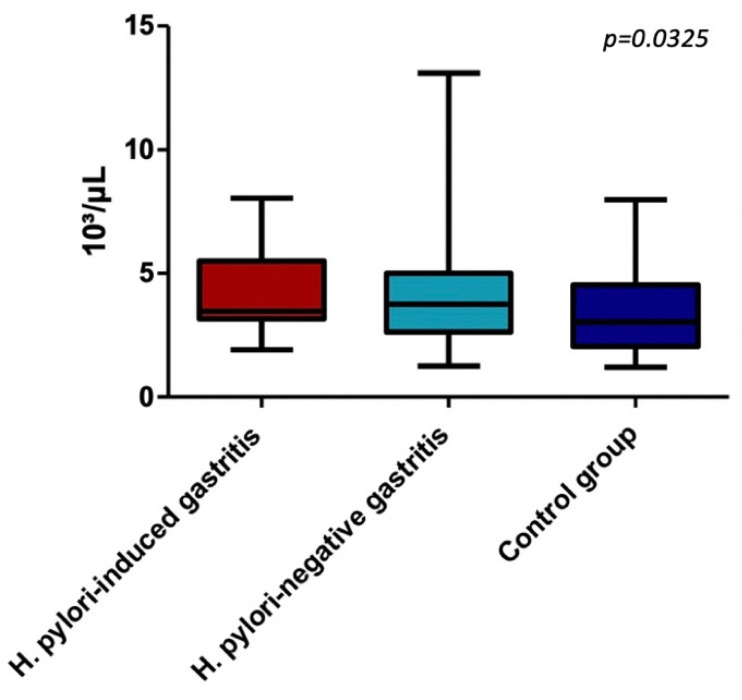
Neutrophils in CT genotype of TLR2 rs3804099 group.

In terms of TLR2 rs380499 gene polymorphism, in the CT variant genotype group, we found a significant difference between median values of neutrophils (*p* = 0.0325), suggesting that CT carriers of this polymorphism had an increased circulating level of neutrophils in the setting of chronic gastric inflammation. Additionally, we noted that the neutrophils in CT genotype groups are significantly higher in *H. pylori*-negative gastritis group versus the control group, *p* = 0.0190. All assessed parameters for each TLR2 rs380499 gene polymorphism are detailed in Table 5 and Table 6, Figure 2.

We also assessed the differences in neutrophil count among each genotype of both polymorphisms TLR2 rs3804099 and NLRP3 rs10754558 for children with *H. pylori*-induced gastritis, but we found no significant differences (Table 7).

Regarding the TLR2 rs380499 gene polymorphism, we found significant differences for CRP values among the three groups in children carrying the TT variant genotype (*p* = 0.0171). Thus, we noticed significantly higher values of CRP values in TT carriers with *H. pylori*-negative gastritis when compared to those with *H. pylori*-induced gastritis (*p* = 0.0264) or healthy controls carrying the same genotype (*p* = 0.0137) (Table 8 and Table 9).

### 3.4. The NLRP3 rs10754558 Gene Polymorphism and Laboratory Parameters

**Figure 3:**
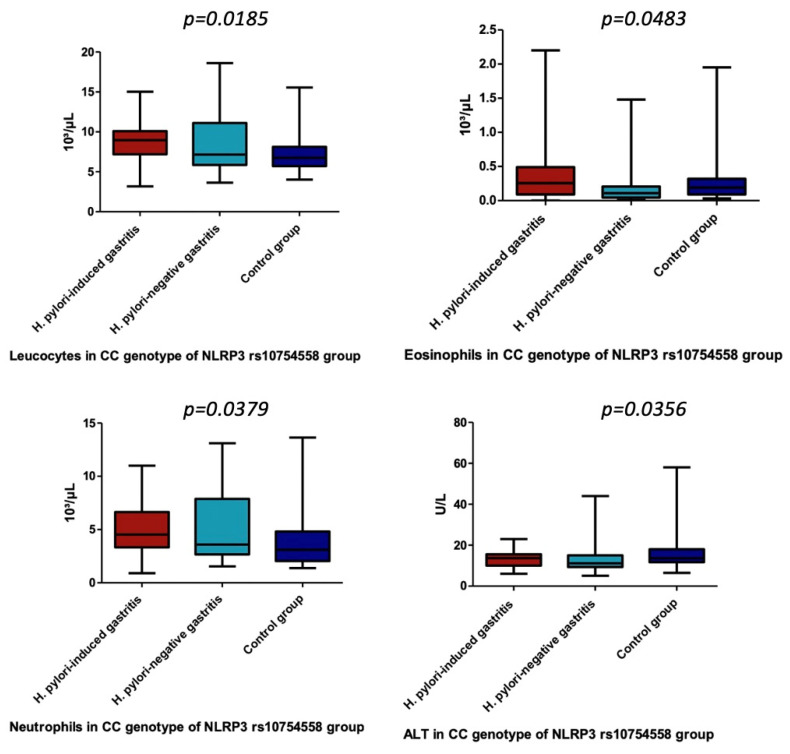
Leucocytes, Eosinophils, Neutrophils, and ALT in CC genotype of NLRP3 rs10754558 group.

Concerning the assessment of NLRP3 rs10754558 gene polymorphisms, we identified significant differences only for CC variant genotype of NLRP3 rs10754558 and leucocytes, neutrophils, eosinophils, as well as ALT (*p* = 0.0185, *p* = 0.0379, *p* = 0.0483, *p* = 0.0356) (Table 10 and Table 11, Figure 3). Therefore, we noticed a significantly higher number of leukocytes in children carrying this genotype diagnosed with *H. pylori*-induced gastritis versus the control group (*p* = 0.0022), while neutrophils in the carriers of the same genotype were significantly higher in *H. pylori*-negative gastritis children (*p* = 0.0151) (Table 11). In addition, we found a significantly higher value for eosinophils in children carrying the CC variant genotype of NLRP3 rs10754558 from *H. pylori*-induced gastritis compared with *H. pylori*-negative gastritis (*p* = 0.0365) (Table 11). The value of ALT was significantly higher in children carrying the previously mentioned genotype from the control group when compared with those included in *H. pylori*-negative gastritis group (*p* = 0.0103) (Table 11).

In terms of NLRP3 rs10754558 gene polymorphism, we found no significant differences for CRP values among the three groups included in the study (Table 12).

## 4. Discussion

*H. pylori* might be defined as ‘the bacterium of childhood’ since it is usually acquired during early childhood, and its prevalence increases with age. Taking into account its close relationship with gastric cancer in adults, detecting factors that prolong its survival within the gastric mucosa from childhood into adulthood promoting the transformation of acute gastritis into chronic inflammation and further into gastric cancer seems to be the missing puzzle piece in terms of strategies meant to decrease the risk of carcinogenesis. Thus, our study aimed to assess children with *H. pylori*-induced gastritis, non-*H. pylori* gastritis, and healthy controls in order to define the role of several environmental, laboratory, and genetic factors that were previously associated either with the development of *H. pylori* infection or with the presence of gastric cancer. In terms of environmental factors, previous studies reported that improper sanitary conditions within the household, bed-sharing between children and adults, along with poor socioeconomic status, increase the risk for *H. pylori* infection [22,23]. Similarly, our findings also pointed out that poor living conditions were significantly associated with *H. pylori*-induced gastritis in children. Moreover, according to a recent study performed on children, originating areas, i.e., rural areas, might also be considered a significant risk factor for developing chronic gastritis, irrespective of the presence of *H. pylori* [24]. Our findings also support the previously noted observation of impact and the correlation between rural areas and the development of gastric inflammation.

TLR2 was strongly related to gastric carcinogenesis. Several studies pointed out that TLR2 polymorphisms are associated with an increased risk for gastric cancer, but this association also depends on ethnicity and geographic area [3,25,26]. Nevertheless, studies on children proved that *H. pylori* has the ability to promote the in vivo overexpression of different TLRs such as TLR2, 4, 5, and 9, early during infection, initiating a chronic and balanced inflammation [27]. This process will continue for decades, defining the pathway towards developing *H. pylori*-related gastropathies during adulthood [20]. Moreover, *H. pylori* infection in children was also associated with an increase in pro and anti-inflammatory cytokines (IL-8, TNF-α, and IL-10) [27]. Pimentel-Nunes et al. noticed an overexpression of TLR2 and TLR4 in intestinal metaplasia and dysplasia/cancer sequence, regardless of the presence of *H. pylori*, but also an upregulation of these TLRs microRNA (mRNA) in individuals with *H. pylori* infection and normal gastric mucosa [28]. These findings suggested that *H. pylori* might not be the only factor that triggers the overexpression of TLRs and the secretion of pro/anti-inflammatory cytokines. These indications are further supported by Targa-Cadamuro et al., who reported an increase in TLR2 and 4 mRNA and protein expression in patients with *H. pylori*-induced chronic gastritis, which persisted even after the eradication therapy [20]. Contrariwise, other TLRs such as TLR10 were proven to ameliorate immune responses in the setting of this infection since their activation resulted in a suppression of proinflammatory cytokines [29], an aspect that requires further studies, especially in pediatric patients. Identifying the activation of TLRs either by *H. pylori* or other PAMPs and damage-associated molecular patterns (DAMPs) fills an important gap in our, still limited, understanding of the pathway towards carcinogenesis and the mechanism of tumor progression [28]. 

Multiple studies assessed the role of TLR2 rs380499 and TLR2 rs384100 gene polymorphisms in patients with *H. pylori* infection, and the results proved to be contradictory. Certain studies performed on patients with gastric cancer revealed a strong association between the previously mentioned SNPs of TLR2 and the risk for gastric carcinogenesis, as well as the prognosis of gastric cancer [30,31]. In contrast, other studies which assessed Japanese, Thai, and Saudi patients with *H. pylori* infection and *H. pylori*-associated gastropathies found no association between either of these two SNPs and the aforementioned conditions [32,33,34]. Similarly, we noticed no association between either TLR2 rs380499 or TLR2 rs384100 gene polymorphisms and the presence of chronic gastritis in children independently of the presence of *H. pylori*. Nevertheless, our study underlined a significant association between the CT variant genotype of TLR2 rs3804099 gene polymorphism and circulating neutrophils in children with non-*H. pylori* gastritis, suggesting that the carriers of this genotype might develop a more severe degree of systemic inflammation, a well-known long-term trigger for carcinogenesis.

NLRP3 is a critical factor for gastric carcinogenesis since it is the well-documented observation that its dependent pathway is crucial for the production of IL-1β in dendritic cells due to *H. pylori* infection [35,36]. Furthermore, IL-1β gene polymorphisms were associated with an increased risk of gastric cancer, while the overexpression of this interleukin resulted in both gastric inflammation and cancer in mice [37,38]. Perez-Figueroa et al. proved that *H. pylori* could increase the expression of NLRP3 inflammasome components, while the inhibitors of this inflammasome and caspase-1 induce a reduction in IL-1β production [6]. In addition, a strong partnership was noted between NLRP3 and TLR2 regarding the production of this interleukin since, according to Jang et al., this TLR is essential for the production of *H. pylori*-induced IL-1β in neutrophils [14]. The same authors noticed a reduction in the expression of both NLRP3 and IL-1β genes in TLR2-deficient neutrophils.

Nevertheless, NLRP3 inflammasome remains the most important host factor in neutrophils that promotes the production of IL-1β in response to *H. pylori* infection since both the secretion and cleavage of this interleukin were abolished in NLRP3- and caspase-1/11-deficient neutrophils [14]. Neutrophils were defined as crucial innate immune cells in *H. pylori*-mediated gastric inflammation [37]. Moreover, they were also related to the development of gastric cancer since an increase in neutrophils recruitment in gastric cancer tissue was reported in comparison with the tissues surrounding the malignant lesion [39,40]. Aside from this local increase in neutrophils related to gastric inflammation, multiple recent studies proved that individuals with *H. pylori*-induced gastritis also present a significant increase in circulating neutrophils, as well as leukocytes, lymphocytes, and acute phase reactants, underlining the ability of this bacterium to trigger a low-grade systemic inflammation [41,42,43,44]. Aside from its suggested role in carcinogenesis, this subclinical inflammation was proved to be associated with several life-threatening chronic conditions such as stroke, cardiovascular diseases, diabetes, thyroid disease, glaucoma, or idiopathic thrombocytopenic purpura [45]. Similar to the findings in TLR2 rs380499 gene polymorphism, we also noticed a significant association between CC variant genotype of NLRP3 rs10754558 gene polymorphism and an increased number of circulating neutrophils in children with *H. pylori*-negative gastritis. Additionally, we found a significant association between this genotype and leukocytes in children diagnosed with *H. pylori*-induced gastritis compared with controls, eosinophils in those with *H. pylori*-induced gastritis compared with those with other types of gastritis, and ALT in the control group versus *H. pylori*-negative gastritis group.

Based on our findings, we might define a trialogue between TLR2, NLRP3, and neutrophils in developing subclinical inflammation related to *H. pylori* infection in children, which might represent an early leading cause for gastric carcinogenesis. Moreover, both bacterial and host factors were proven to have a synergistic role in the production of IL-1β in neutrophils [14].

The main limitation of this study consists in the relatively small number of children diagnosed with H. pylori-induced chronic gastritis that might have led to the lack of correlation between the assessed gene polymorphisms and the presence of H. pylori. The fact that we included children originating from a single area of Romania could represent another limitation since we noticed that both ethnicity and geographic area play a crucial role in the development of H. pylori-related gastropathies. It is also worth mentioning that we did not use validated tools for defining living conditions, which may be seen as another possible study limitation. Furthermore, it would have been extremely useful to assess our sample after the eradication therapy for this infection, but unfortunately, once the symptoms disappeared, the patients did not return for the follow-up. Nevertheless, several valuable strengths of this study should be emphasized: in our study, a significant number of pediatric patients were accurately diagnosed based on gastric biopsy specimens, unlike most studies reported in the literature where pediatric age implied only noninvasive methods. We assessed three gene polymorphisms previously reported to be crucial for gastric carcinogenesis, and we also included both patients with *H. pylori*-positive and *H. pylori*-negative gastritis in comparison with those with the normal gastric mucosa. To the best of our knowledge, ours is among the few studies, if not the first reported in the literature to assess three gene polymorphisms, i.e., *TLR2* rs3804099, *TLR2* rs3804100, and *NLRP3* rs10754558, in children with *H. pylori*-induced gastritis, *H. pylori*-negative gastritis, and *H. pylori*-negative normal gastric mucosa in order to underline the early possible triggers of gastric carcinogenesis.

## 5. Conclusions

Our findings indicated that poor living conditions and rural areas might be considered important risk factors for developing pediatric chronic gastritis, whether induced by *H. pylori* infection or not. Rapid urease test proved to be reliable for the early detection of *H. pylori* infection. Our study revealed that both CT and TT carriers of *TLR2* rs3804099 gene polymorphism might display a more severe degree of systemic inflammation considering their increased level of circulating neutrophils and CRP in the setting of gastric inflammation. Similarly, the CC genotype carriers of NLRP3 rs10754558 polymorphism detected with *H. pylori*-negative gastritis were also found with significantly increased levels of circulating neutrophils. Moreover, these carriers associated with significantly higher circulating levels of leukocytes and eosinophils in the setting of *H. pylori*-induced gastritis. Therefore, children with gastritis carrying NLRP3 rs10754558 polymorphism might have an increased risk for developing subclinical systemic inflammation, irrespective of the presence of *H. pylori*. The significant association between CC genotype of NLRP3 rs10754558 polymorphism and ALT in children with *H. pylori*-negative gastritis suggests a possible susceptibility of these carriers to associate liver impairment in the context of gastric inflammation. Taking into account the association between both TLR2 rs380499 and NLRP3 rs10754558 gene polymorphisms and increased level of circulating neutrophils, also known as the key innate immune cells, involved in both local gastric and systemic inflammation, it is conceivable that host genetic factors are crucial for the development of *H. pylori* and non-*H. pylori*-induced gastropathies. These findings represent a solid basis for further studies to determine the precise role of these polymorphisms in children with *H. pylori*- and non-*H. pylori*-gastritis.

## Figures and Tables

**Table 1:** The demographic analysis of the three groups.

Parameters	*H. pylori*-Induced Gastritis (*n* = 51)	*H. pylori*-Negative Gastritis (*n* = 103)	Control Group (*n* = 115)	*p* Value
Age (years)				
	12.92 ± 3.783 (13.00)	12.15 ± 4.514 (13.00)	12.14 ± 3.783 (13.00)	* 0.4892
Gender				
Girls	32 (62.75%)	68 (66.02%)	68 (59.13%)	0.5764
Boys	19 (37.25%)	35 (33.98%)	47 (40.87%)	
Originating Area				
Urban	17 (33.33%)	45 (43.69%)	66 (57.39%)	0.0100
Rural	34 (66.67%)	58 (56.31%)	49 (42.61%)	
TLR2 rs3804099				
CT genotype	16 (31.37%)	53 (51.46%)	54 (46.96%)	0.1339
TT genotype	27 (52.94%)	33 (32.04%)	43 (37.39%)	
CC genotype	8 (15.69%)	17 (16.50%)	18 (15.65%)	
TLR2 rs3804100				
CT genotype	3 (5.88%)	14 (13.59%)	17 (14.78%)	0.5570
TT genotype	47 (92.16%)	88 (85.44%)	97 (84.35%)	
CC genotype	1 (1.96%)	1 (0.97%)	1 (0.87%)	
NLRP3 rs10754558				
CC genotype	20 (39.22%)	37 (35.92%)	43 (37.39%)	0.5971
CG genotype	27 (52.94%)	50 (48.55%)	52 (45.22%)	
GG genotype	4 (7.84%)	16 (15.53%)	20 (17.39%)	
Family History				
Absent	50 (98.04%)	101 (98.06%)	114 (99.13%)	0.7700
Present	1 (1.96%)	2 (1.94%)	1 (0.87%)	
Living Conditions				
Good	33 (64.71%)	89 (86.41%)	107 (93.04%)	<0.0001
Poor	18 (35.29%)	14 (13.59%)	8 (6.96%)	
IgA Anti-H. pylori				
Positive	34 (66.67%)	7 (6.80%)	5 (4.35%)	<0.0001
Negative	17 (33.33%)	96 (93.20%)	110 (95.65%)	
Gastroesophageal Reflux				
Present	9 (17.65%)	19 (18.45%)	30 (26.09%)	0.2944
Absent	42 (82.35%)	84 (81.55%)	85 (73.91%)	
Biliary Reflux				
Present	12 (23.53%)	35 (33.98%)	35 (30.43%)	0.4151
Absent	39 (76.47%)	68 (66.02%)	80 (69.57%)	
Rapid Urease Test				
Positive	34 (66.67%)	13 (12.62%%)	15 (13.04%)	<0.0001
Negative	17 (33.33%)	86 (83.50%)	99 (86.09%)	

Legend: *n*—number, TLR2 rs3804099 TT—homozygous wild-type genotype, CC—homozygous variant genotype, TLR2 rs3804100 TT—homozygous wild-type genotype, CC—homozygous variant genotype, NLRP3 rs10754558 GG—homozygous wild-type genotype, CC—homozygous variant genotype, * Kruskal–Wallis test.

**Table 2:** The assessment of the laboratory parameters between the three groups.

Parameters	*H. pylori*-Induced Gastritis (*n* = 51)	*H. pylori*-Negative Gastritis (*n* = 103)	Control Group (*n* = 115)	*p*-Value
Hemoglobin (g/dL)	13.50 ± 2.101 (13.20)	13.31 ± 1.244 (13.30)	13.44 ± 1.373 (13.50)	* 0.7911
Leucocytes (10^3^/µL)	7.844 ± 2.282 (7.55)	7.833 ± 2.773 (7.00)	7.200 ± 2.457 (6.69)	* 0.0527
Lymphocytes (10^3^/µL)	2.482 ± 0.6812 (2.50)	2.579 ± 0.8888 (2.50)	2.434 ± 0.7767 (2.42)	* 0.4405
Neutrophils (10^3^/µL)	4.367 ± 2.096 (3.85)	4.298 ± 2.471 (3.60)	3.905 ± 2.353 (3.32)	* 0.1313
Eosinophils (10^3^/µL)	0.2773 ± 0.3965 (0.12)	0.2527 ± 0.8225 (0.136)	0.2236 ± 0.2984 (0.11)	* 0.7122
ESR (mm/h)	11.00 ± 9.302 (9.00)	10.89 ± 9.555 (7.00)	9.496 ± 7.315 (7.00)	* 0.5730
Iron (µmol/L)	15.27 ± 7.007 (14.22)	14.80 ± 7.682 (12.97)	15.68 ± 6.951 (14.94)	* 0.3804
AST (U/L)	20.89 ± 6.705 (19.60)	22.27 ± 9.594 (18.70)	22.91 ± 11.01 (20.20)	* 0.5924
ALT (U/L)	12.95 ± 4.269 (12.20)	14.48 ± 10.07 (12.00)	14.75 ± 7.305 (13.10)	* 0.1189
GGT (U/L)	11.51 ± 3.313 (11.00)	13.31 ± 7.750 (11.00)	12.97 ± 4.535 (12.00)	* 0.1004
NLR	1.985 ± 1.445 (1.43)	2.053 ± 1.866 (1.44)	1.859 ± 1.589 (1.35)	* 0.5609

Legend: AST = aspartate aminotransferase, ALT = alanine aminotransferase, GGT = gamma-glutamyl transpeptidase, NLR = neutrophils/lymphocytes ratio, estimated significance value obtained from nonparametric Mann–Whitney test; * Kruskal–Wallis test.

**Table 3:** The assessment of CRP between the three groups.

Parameters	*H. pylori*-Induced Gastritis (*n* = 27)	*H. pylori*-Negative Gastritis (*n* = 70)	Control Group (*n* = 69)	*p*-Value
**CRP** (mg/L)	0.6048 ± 0.8973 (0.21)	1.911 ± 3.456 (0.435)	1.157 ± 2.400 (0.20)	* **0.0452**

Legend: CRP = C reactive protein; * Kruskal-Wallis test.

**Table 4:** The difference of median values of CRP between the three groups.

	Dunn’s Multiple Comparison Test	Significant? *p* < 0.05?
**CRP** (mg/L)	*H. pylori*-induced gastritis vs. *H. pylori*-negative gastritis	0.0839
	*H. pylori*-induced gastritis vs. control group	0.5880
	*H. pylori*-negative gastritis vs. control group	* **0.0232**

Legend: CRP = C reactive protein; * Kruskal–Wallis test.

**Table 5:** The assessment of the laboratory parameters between the three groups among TLR2 rs380499 gene polymorphism types.

**CC Genotype of *TLR2* rs3804099 (*n* = 43)**	***H. pylori*-Induced Gastritis (*n* = 8) Mean ± SD (Median)**	***H. pylori*-Negative Gastritis (*n* = 17) Mean ± SD (Median)**	**Control Group (*n* = 18) Mean ± SD (Median)**	***p*-Value**
Hemoglobin (g/dL)	14.10 ± 1.298 (13.95)	13.72 ± 1.103 (13.50)	13.49 ± 1.228 (13.75)	* 0.8040
Leucocytes (10^3^/µL)	7.223 ± 1.122 (7.29)	7.473 ± 1.212 (7.03)	8.607 ± 3.842 (6.735)	* 0.9600
Lymphocytes (10^3^/µL)	2.436 ± 0.6104 (2.65)	2.941 ± 0.8782 (2.75)	2.427 ± 0.7667 (2.335)	0.1293
Neutrophils (10^3^/µL)	3.983 ± 0.9837 (3.82)	3.706 ± 1.324 (3.58)	5.237 ± 3.838 (3.88)	* 0.5713
Eosinophils (10^3^/µL)	0.1450 ± 0.1574 (0.075)	0.1765 ± 0.1198 (0.16)	0.1728 ± 0.2815 (0.075)	* 0.2337
ESR (mm/h)	8.125 ± 5.194 (6.00)	10.12 ± 9.151 (6.00)	11.67 ± 8.918 (9.00)	* 0.4078
Iron µmol/L)	15.76 ± 4.594 (15.40)	17.44 ± 9.488 (13.90)	14.88 ± 6.415 (14.98)	0.6059
AST (U/L)	21.90 ± 4.524 (21.85)	20.41 ± 9.228 (18.00)	20.97 ± 5.388 (19.25)	* 0.3246
ALT (U/L)	13.24 ± 6.625 (10.35)	14.07 ± 8.557 (12.40)	15.68 ± 6.468 (14.55)	* 0.1713
GGT (U/L)	12.50 ± 4.408 (11.00)	11.76 ± 4.085 (11.00)	12.33 ± 3.361 (11.50)	* 0.5595
NLR	1.769 ± 0.7707 (1.34)	1.439 ± 0.8441 (1.14)	2.515 ± 2.293 (1.845)	* 0.1417
**CT Genotype of *TLR2* rs3804099 (*n* = 123)**	***H. pylori*-Induced Gastritis (*n* = 16) Mean ± SD (Median)**	***H. pylori*-Negative Gastritis (*n* = 53) Mean ± SD (Median)**	**Control Group (*n* = 54) Mean ± SD (Median)**	***p*-Value**
Hemoglobin (g/dL)	13.79 ± 3.279 (13.45)	13.34 ± 1.262 (13.40)	13.25 ± 1.252 (13.20)	* 0.6544
Leucocytes (10^3^/µL)	7.833 ± 1.975 (7.56)	7.942 ± 3.043 (6.97)	6.850 ± 1.914 (6.515)	* 0.1271
Lymphocytes (10^3^/µL)	2.509 ± 0.7732 (2.515)	2.486 ± 0.8020 (2.48)	2.570 ± 0.8400 (2.53)	* 0.9816
Neutrophils (10^3^/µL)	4.273 ± 1.795 (3.465)	4.402 ± 2.589 (3.76)	3.385 ± 1.607 (3.035)	* 0.0325
Eosinophils (10^3^/µL)	0.3044 ± 0.3601 (0.18)	0.3216 ± 1.128 (0.12)	0.2767 ± 0.3675 (0.13)	* 0.3403
ESR (mm/h)	11.13 ± 6.642 (10.00)	10.85 ± 9.183 (7.00)	8.093 ± 5.307 (6.00)	* 0.1487
Iron µmol/L)	16.79 ± 9.143 (14.32)	14.01 ± 6.380 (12.07)	16.49 ± 7.274 (15.95)	* 0.1597
AST (U/L)	22.36 ± 7.181 (20.60)	21.19 ± 7.102 (18.40)	23.98 ± 13.86 (20.80)	* 0.4587
ALT (U/L)	13.01 ± 3.613 (11.95)	14.55 ± 11.54 (12.40)	14.88 ± 7.692 (12.95)	* 0.4954
GGT (U/L)	11.63 ± 3.828 (10.50)	13.42 ± 8.423 (11.00)	13.07 ± 4.421 (12.00)	* 0.4162
NLR	1.996 ± 1.455 (1.525)	2.157 ± 2.009 (1.57)	1.455 ± 0.9200 (1.14)	* 0.0676
**TT Genotype of *TLR2* rs3804099 (*n* = 103)**	***H. pylori*-Induced Gastritis (*n* = 27) Mean ± SD (Median)**	***H. pylori*-Negative Gastritis (*n* = 33) Mean ± SD (Median)**	**Control Group (*n* = 43) Mean ± SD (Median)**	***p*-Value**
Hemoglobin (g/dL)	13.16 ± 1.241 (13.00)	13.05 ± 1.256 (13.20)	13.65 ± 1.561 (13.60)	* 0.1838
Leucocytes (10^3^/µL)	8.035 ± 2.696 (7.74)	7.844 ± 2.938 (7.00)	7.051 ± 2.181 (6.78)	* 0.2410
Lymphocytes (10^3^/µL)	2.479 ± 0.6679 (2.35)	2.542 ± 1.001 (2.34)	2.266 ± 0.6753 (2.38)	0.2859
Neutrophils (10^3^/µL)	4.537 ± 2.499 (4.12)	4.435 ± 2.734 (3.36)	4.002 ± 2.166 (3.48)	* 0.7447
Eosinophils (10^3^/µL)	0.3013 ± 0.4655 (0.125)	0.1814 ± 0.2553 (0.136)	0.1728 ± 0.1801 (0.11)	* 0.6806
ESR (mm/h)	11.78 ± 11.42 (9.00)	11.36 ± 10.56 (8.00)	10.35 ± 8.499 (9.00)	* 0.9502
Iron µmol/L)	14.22 ± 6.154 (12.06)	14.71 ± 8.498 (13.41)	15.00 ± 6.792 (12.29)	* 0.8902
AST (U/L)	19.73 ± 6.938 (18.00)	24.97 ± 12.55 (22.30)	22.38 ± 8.432 (19.60)	* 0.1835
ALT (U/L)	12.83 ± 3.956 (12.90)	14.59 ± 8.361 (11.20)	14.19 ± 7.247 (13.10)	* 0.6592
GGT (U/L)	11.15 ± 2.641 (11.00)	13.94 ± 8.131 (12.00)	13.12 ± 5.137 (11.00)	* 0.1578
NLR	2.043 ± 1.618 (1.54)	2.201 ± 1.987 (1.44)	2.093 ± 1.791 (1.60)	* 0.9275

Legend: AST = aspartate aminotransferase, ALT = alanine aminotransferase, ESR = erythrocyte sedimentation rate, GGT = gamma-glutamyl transpeptidase, NLR = neutrophils/lymphocytes ratio, estimated significance value obtained from nonparametric Mann–Whitney test; TT—homozygous wild genotype, CT—heterozygous variant genotype, CC—homozygous variant genotype, * Kruskal–Wallis test.

**Table 6:** The difference of median values of neutrophils between the three groups in CT variant genotype of TLR2 rs3804099 group.

	Dunn’s Multiple Comparison Test	Significant? *p* < 0.05?
Neutrophils in CT genotype group	*H. pylori*-induced gastritis vs. *H. pylori*-negative gastritis	0.7383
	*H. pylori*-induced gastritis vs. control group	0.0639
	*H. pylori*-negative gastritis vs. control group	0.0190

Legend: CT—heterozygous variant genotype, Kruskal–Wallis test.

**Table 7:** The difference in neutrophils counts in *H. pylori*-induced gastritis groups according to various genotypes.

***H. pylori*-induced gastritis (*n* = 51)**	**CC Genotype of TLR2 rs3804099 (*n* = 8)** **Mean ± SD (Median)**	**CT Genotype of TLR2 rs3804099 (*n* = 16)** **Mean ± SD (Median)**	**TT Genotype of TLR2 rs3804099 (*n* = 27)** **Mean ± SD (Median)**	***p*-Value**
Neutrophils **(10^3^/µL)**	3.983 ± 0.9837 (3.820)	4.273 ± 1.795 (3.465)	4.537 ± 2.499 (4.12)	* 0.9840
***H. pylori*-Induced Gastritis (*n* = 51)**	**CC Genotype of NLRP3 rs10754558 (*n* = 20)** **Mean ± SD (Median)**	**CG Genotype of NLRP3 rs10754558 (*n* = 27)** **Mean ± SD (Median)**	**GG Genotype of NLRP3 rs10754558 (*n* = 3)** **Mean ± SD (Median)**	***p*-Value**
Neutrophils **(10^3^/µL)**	4.939 ± 2.417 (4.53)	3.863 ± 1.705 (3.27)	4.908 ± 2.481 (3.895)	* 0.1595

Legend: TT—homozygous wild genotype, CT—heterozygous variant genotype, CC—homozygous variant genotype of TLR2 rs380499 gene polymorphism types; GG—homozygous wild genotype, GC—heterozygous variant genotype, CC—homozygous variant genotype of NLRP3 rs10754558 gene polymorphism types, * Kruskal–Wallis test.

**Table 8:** The assessment of the CRP between the three groups among TLR2 rs380499 gene polymorphism types.

**CC Genotype of *TLR2* rs3804099 (*n* = 28)**	***H. pylori*-Induced Gastritis (*n* = 7) Mean ± SD (Median)**	***H. pylori*-Negative Gastritis (*n* = 10) Mean ± SD (Median)**	**Control Group (*n* = 11) Mean ± SD (Median)**	***p*-Value**
CRP (mg/L)	1.003 ± 1.436 (0.40)	0.1640 ± 0.1414 (0.20)	1.998 ± 4.471 (0.20)	* 0.2366
**CT Genotype of *TLR2* rs3804099 (*n* = 74)**	***H. pylori*-Induced Gastritis (*n* = 8) Mean ± SD (Median)**	***H. pylori*-Negative Gastritis (*n* = 34) Mean ± SD (Median)**	**Control Group (*n* = 32) Mean ± SD (Median)**	***p*-Value**
CRP (mg/L)	0.4013 ± 0.5608 (0.215)	2.309 ± 3.803 (0.39)	1.113 ± 2.116 (0.20)	* 0.0833
**TT Genotype of *TLR2* rs3804099 (*n* = 63)**	***H. pylori*-Induced Gastritis (*n* = 12) Mean ± SD (Median)**	***H. pylori*-Negative Gastritis (*n* = 26) Mean ± SD (Median)**	**Control Group (*n* = 25) Mean ± SD (Median)**	***p*-Value**
CRP (mg/L)	0.5083 ± 0.6606 (0.205)	2.063 ± 3.523 (1.020)	0.7676 ± 1.255 (0.20)	* 0.0171

Legend: CRP = C reactive protein, estimated significance value obtained from nonparametric Mann–Whitney test; TT—homozygous wild genotype, CT—heterozygous variant genotype, CC—homozygous variant genotype, * Kruskal-Wallis test.

**Table 9:** The difference of median values of CRP between the three groups in TT variant genotype of TLR2 rs3804099 group.

	Dunn’s Multiple Comparison Test	Significant? *p* < 0.05?
CRP in TT genotype group	*H. pylori*-induced gastritis vs. *H. pylori*-negative gastritis	0.0264
	*H. pylori*-induced gastritis vs. control group	0.4797
	*H. pylori*-negative gastritis vs. control group	0.0137

Legend: CRP = C reactive protein, TT—homozygous wild genotype, Kruskal–Wallis test.

**Table 10:** The assessment of the laboratory parameters between the three groups among NLRP3 rs10754558 gene polymorphism types.

**CC Genotype of *NLRP3* rs10754558 (*n* = 100)**	***H. pylori*-Induced Gastritis (*n* = 20) Mean ± SD (Median)**	***H. pylori*-Negative Gastritis (*n* = 37) Mean ± SD (Median)**	**Control Group (*n* = 43) Mean ± SD (Median)**	***p*-Value**
Hemoglobin (g/dL)	13.44 ± 0.9539 (13.15)	13.36 ± 1.113 (13.30)	13.52 ± 1.137 (13.60)	* 0.8610
Leucocytes (10^3^/µL)	8.741 ± 2.595 (8.955)	8.508 ± 3.778 (7.16)	7.055 ± 1.956 (6.76)	* 0.0185
Lymphocytes (10^3^/µL)	2.616 ± 0.7164 (2.575)	2.459 ± 0.9214 (2.50)	2.528 ± 0.6515 (2.44)	* 0.7465
Neutrophils (10^3^/µL)	4.939 ± 2.417 (4.53)	5.116 ± 3.432 (3.60)	3.630 ± 2.058 (3.11)	* 0.0379
Eosinophils (10^3^/µL)	0.4080 ± 0.5380 (0.255)	0.1815 ± 0.2561 (0.11)	0.2444 ± 0.3119 (0.19)	* 0.0483
ESR (mm/h)	11.60 ± 8.426 (9.50)	11.59 ± 10.43 (7.00)	9.953 ± 7.946 (7.00)	* 0.8556
Iron (µmol/L)	13.76 ± 4.664 (12.26)	14.87 ± 9.315 (13.21)	17.31 ± 7.581 (15.86)	* 0.0518
AST (U/L)	20.82 ± 6.462 (19.55)	21.59 ± 6.499 (19.50)	24.56 ± 8.946 (22.50)	* 0.2203
ALT (U/L)	13.33 ± 4.149 (13.70)	13.45 ± 8.060 (11.20)	16.54 ± 9.687 (13.60)	* 0.0356
GGT (U/L)	11.25 ± 3.193 (11.00)	13.76 ± 9.023 (12.00)	12.81 ± 5.491 (11.00)	* 0.4598
NLR	2.061 ± 1.314 (1.79)	2.577 ± 2.457 (1.32)	1.655 ± 1.621 (1.28)	* 0.1338
**CC Genotype of *NLRP3* rs10754558 (*n* = 129)**	***H. pylori*-Induced Gastritis (*n* = 27) Mean ± SD (Median)**	***H. pylori*-Negative Gastritis (*n* = 50) Mean ± SD (Median)**	**Control Group (*n* = 52) Mean ± SD (Median)**	***p*-Value**
Hemoglobin (g/dL)	13.59 ± 2.782 (13.50)	13.33 ± 1.367 (13.45)	13.31 ± 1.506 (13.40)	* 0.9528
Leucocytes (10^3^/µL)	7.161 ± 1.822 (6.81)	7.397 ± 2.010 (6.945)	6.920 ± 2.444 (6.515)	* 0.2235
Lymphocytes (10^3^/µL)	2.391 ± 0.6906 (2.42)	2.603 ± 0.8177 (2.425)	2.417 ± 0.8300 (2.425)	* 0.5341
Neutrophils (10^3^/µL)	3.863 ± 1.705 (3.27)	3.794 ± 1.434 (3.615)	3.672 ± 2.126 (3.305)	* 0.4342
Eosinophils (10^3^/µL)	0.2039 ± 0.2466 (0.10)	0.3278 ± 1.158 (0.145)	0.2306 ± 0.3197 (0.105)	* 0.7530
ESR (mm/h)	10.48 ± 10.60 (7.00)	10.30 ± 8.572 (7.50)	8.558 ± 6.446 (6.00)	* 0.5342
Iron (µmol/L)	17.01 ± 8.415 (15.78)	14.21 ± 6.845 (11.88)	14.93 ± 6.735 (13.15)	* 0.2395
AST (U/L)	20.74 ± 6.604 (20.80)	21.77 ± 7.457 (19.90)	22.78 ± 13.65 (20.15)	* 0.9339
ALT (U/L)	12.94 ± 4.535 (11.70)	15.40 ± 11.81 (12.45)	13.53 ± 4.766 (12.95)	* 0.5719
GGT (U/L)	11.78 ± 3.672 (11.00)	13.50 ± 7.451 (11.50)	13.06 ± 4.161 (12.00)	* 0.3507
NLR	1.908 ± 1.592 (1.37)	1.748 ± 1.369 (1.445)	1.721 ± 1.319 (1.325)	* 0.8565
**GG Genotype of *NLRP3* rs10754558 (*n* = 40)**	***H. pylori*-Induced Gastritis (*n* = 4) Mean ± SD (Median)**	***H. pylori*-Negative Gastritis (*n* = 16) Mean ± SD (Median)**	**Control Group (*n* = 20) Mean ± SD (Median)**	***p*-Value**
Hemoglobin (g/dL)	13.25 ± 0.7188 (13.00)	13.13 ± 1.183 (13.00)	13.59 ± 1.514 (13.65)	* 0.3968
Leucocytes (10^3^/µL)	7.973 ± 2.405 (7.14)	7.634 ± 1.737 (7.49)	8.240 ± 3.213 (7.21)	* 0.9920
Lymphocytes (10^3^/µL)	2.420 ± 0.3879 (2.305)	2.783 ± 1.034 (2.66)	2.276 ± 0.8859 (2.09)	* 0.3517
Neutrophils (10^3^/µL)	4.908 ± 2.481 (3.895)	3.983 ± 1.987 (3.60)	5.105 ± 3.146 (3.975)	* 0.4413
Eosinophils (10^3^/µL)	0.06667 ± 0.03512 (0.07)	0.1829 ± 0.1587 (0.138)	0.1605 ± 0.1991 (0.08)	* 0.1685
ESR (mm/h)	11.50 ± 3.697 (11.50)	11.13 ± 10.83 (8.00)	10.95 ± 8.056 (8.50)	* 0.5106
Iron µmol/L)	11.02 ± 0.4034 (11.11)	16.48 ± 5.981 (16.36)	14.10 ± 5.564 (12.25)	* 0.1168
AST (U/L)	22.33 ± 10.19 (18.45)	25.41 ± 18.18 (17.15)	19.69 ± 5.672 (19.05)	* 0.9596
ALT (U/L)	11.13 ± 3.327 (10.00)	13.99 ± 8.463 (11.80)	14.08 ± 6.324 (12.25)	* 0.4787
GGT (U/L)	11.00 ± 0.00 (11.00)	11.69 ± 5.313 (10.50)	13.10 ± 3.194 (12.00)	* 0.0811
NLR	2.128 ± 1.331 (1.55)	1.793 ± 1.418 (1.525)	2.659 ± 1.963 (2.225)	* 0.2750

Legend: AST = aspartate aminotransferase, ALT = alanine aminotransferase, ESR—erythrocyte sedimentation rate, GGT = gamma-glutamyl transpeptidase, NLR = neutrophils/lymphocytes ratio, estimated significance value obtained from nonparametric Mann–Whitney test; GG—homozygous wild genotype, GC—heterozygous variant genotype, CC—homozygous variant genotype, * Kruskal–Wallis test.

**Table 11:** The difference of median values of leucocytes, neutrophils, eosinophils, and ALT between the three groups in CC variant genotype of NLRP3 rs10754558 group.

	Dunn’s Multiple Comparison Test	Significant? *p* < 0.05?
Leucocytes in CC genotype group	*H. pylori*-induced gastritis vs. *H. pylori*-negative gastritis	0.2129
	*H. pylori*-induced gastritis vs. control group	0.0022
	*H. pylori*-negative gastritis vs. control group	0.2132
Neutrophils in CC genotype group	*H. pylori*-induced gastritis vs. *H. pylori*-negative gastritis	0.4877
	*H. pylori*-induced gastritis vs. control group	0.0774
	*H. pylori*-negative gastritis vs. control group	0.0151
Eosinophils in CC genotype group	*H. pylori*-induced gastritis vs. *H. pylori*-negative gastritis	0.0365
	*H. pylori*-induced gastritis vs. control group	0.3754
	*H. pylori*-negative gastritis vs. control group	0.0516
ALT in CC genotype group	*H. pylori*-induced gastritis vs. *H. pylori*-negative gastritis	0.2921
	*H. pylori*-induced gastritis vs. control group	0.2911
	*H. pylori*-negative gastritis vs. control group	**0.0103**

Legend: ALT = alanine aminotransferase, CC—homozygous variant genotype, Kruskal-Wallis test.

**Table 12:** The assessment of the CRP between the three groups among NLRP3 rs10754558 gene polymorphism types.

**CC Genotype of NLRP3 rs10754558 (*n* = 60)**	***H. pylori*-Induced Gastritis (*n* = 8) Mean ± SD (Median)**	***H. pylori*-Negative Gastritis (*n* = 26) Mean ± SD (Median)**	**Control Group (*n* = 26) Mean ± SD (Median)**	***p*-Value**
CRP (mg/L)	0.8288 ± 0.8528 (0.315)	3.140 ± 5.131 (0.515)	1.052 ± 2.003 (0.20)	* 0.1133
**CC Genotype of NLRP3 rs10754558 (*n* = 83)**	***H. pylori*-Induced Gastritis (*n* = 16) Mean ± SD (Median))**	***H. pylori*-Negative Gastritis (*n* = 33) Mean ± SD (Median)**	**Control Group (*n* = 34) Mean ± SD (Median)**	***p*-Value**
CRP (mg/L)	0.545 ± 1.004 (0.20)	1.131 ± 1.513 (0.38)	0.8482 ± 1.548 (0.24)	* 0.1574
**CC Genotype of NLRP3 rs10754558 (*n* = 22)**	***H. pylori*-Induced Gastritis (*n* = 3) Mean ± SD (Median)**	***H. pylori*-Negative Gastritis (*n* = 11) Mean ± SD (Median)**	**Control Group (*n* = 8) Mean ± SD (Median)**	***p*-Value**
CRP (mg/L)	0.3267 ± 0.1137 (0.36)	1.350 ± 1.732 (0.25)	2.576 ± 5.196 (0.35)	* 0.9224

Legend: CRP = C reactive protein, estimated significance value obtained from nonparametric Mann–Whitney test; GG—homozygous wild genotype, GC—heterozygous variant genotype, CC—homozygous variant genotype, * Kruskal–Wallis test.

## Data Availability

Not applicable.
